# Enhanced Nitrogen Availability in Karst Ecosystems by Oxalic Acid Release in the Rhizosphere

**DOI:** 10.3389/fpls.2016.00687

**Published:** 2016-05-24

**Authors:** Fujing Pan, Yueming Liang, Wei Zhang, Jie Zhao, Kelin Wang

**Affiliations:** ^1^Key Laboratory of Agro-ecological Processes in Subtropical Region, Institute of Subtropical Agriculture, Chinese Academy of SciencesChangsha, China; ^2^University of Chinese Academy of SciencesBeijing, China; ^3^Huanjiang Observation and Research Station for Karst EcosystemsHuanjiang, China; ^4^Karst Dynamics Laboratory, Ministry of Land and Resources, Institute of Karst Geology, Chinese Academy of Geological SciencesGuilin, China

**Keywords:** β-1,4-*N*-acetylglucosaminidase, karst shrubs and trees, microbial biomass, oxalic acid, potential N mineralization rates

## Abstract

In karst ecosystems, a high level of CaCO_3_ enhances the stabilization of soil organic matter (SOM) and causes nitrogen (N) and/or phosphorus (P) limitation in plants. Oxalic acid has been suggested to be involved in the nutrient-acquisition strategy of plants because its addition can temporarily relieve nutrient limitation. Therefore, understanding how oxalic acid drives N availability may help support successful vegetation restoration in the karst ecosystems of southwest China. We tested a model suggested by Clarholm et al. (2015) where oxalate reacts with Ca bridges in SOM, thus exposing previously protected areas to enzymatic attacks in a way that releases N for local uptake. We studied the effects of oxalic acid, microbial biomass carbon (MBC), and β-1,4-*N*-acetylglucosaminidase (NAG) on potential N mineralization rates in rhizosphere soils of four plant species (two shrubs and two trees) in karst areas. The results showed that rhizosphere soils of shrubs grown on formerly deforested land had significantly lower oxalic acid concentrations and NAG activity than that of trees in a 200-year-old forest. The levels of MBC in rhizosphere soils of shrubs were significantly lower than those of trees in the growing season, but the measure of shrubs and trees were similar in the non-growing season; the potential N mineralization rates showed a reverse pattern. Positive relationships were found among oxalic acid, MBC, NAG activity, and potential N mineralization rates for both shrubs and trees. This indicated that oxalic acid, microbes, and NAG may enhance N availability for acquisition by plants. Path analysis showed that oxalic acid enhanced potential N mineralization rates indirectly through inducing microbes and NAG activities. We found that the exudation of oxalic acid clearly provides an important mechanism that allows plants to enhance nutrient acquisition in karst ecosystems.

## Introduction

Karst landscapes are distributed worldwide and account for nearly 15% of Earth’s land surface. The karst region of southwestern China, one of the world’s largest karst regions, encompasses 540,000 km^2^ (Yuan, 1994; Qi et al., 2013). Characteristics of this region include a high ratio of rocky exposure, alkaline calcareous soils, and specialized regional vegetation (Su and Li, 2003; Chen et al., 2010; Zhang et al., 2012). The availability of soil nitrogen (N) and phosphorus (P) are alternatively limiting elements for primary productivity in karst ecosystems (Niinemets and Kull, 2005; Pan et al., 2015; Zhang et al., 2015). N and P are mainly released for plant uptake from the decomposition of soil organic matter (SOM) (Bertrand et al., 2006; Dijkstra et al., 2009). Large fractions of N and P are stabilized in karst soils by becoming directly bound to Ca minerals and by numerous Ca bridges in SOM (Hu et al., 2012; Clarholm et al., 2015), resulting in low N and P availability (Strom et al., 2005). Additionally, newly dissolved nutrients are easily leached into underground drainage networks caused by the shallow soil cover of karst ecosystems and the highly developed epikarst system (Zhang et al., 2011), which exacerbate nutrient limitation in these systems.

To adapt to the nutrient-limited conditions, plants have developed a wide variety of nutrient acquisition strategies. They include: (1) genome size evolution of many plants that enhances DNA and protein building blocks needed for growth (Kang et al., 2015); (2) resorption of nutrients from senescent foliage that decreases nutrients loss (Du et al., 2011); (3) symbiosis of roots with arbuscular mycorrhizal fungi that enhances soil weathering (Thorley et al., 2014; Liang et al., 2015), or symbiosis with N fixing bacteria, which bind N from the atmosphere (Liu et al., 2015a); and (4) priming by fine roots and mycorrhizal hyphae, e.g., exudation of organic acids and enzymes into rhizosphere soils, a process that will enhance SOM decomposition and nutrients release (Strom et al., 2005; Clarholm et al., 2015).

Shrubland is widely distributed in the karst region of China and represents a typical transitional stage between grassland and primary forest in formerly deforested land. Researchers have suggested using the shrub stage as the first choice for vegetation restoration in this region (Pan et al., 2015) by planting supplementary shrubs. Our previous study indicated that shrubs in the shrub stage of ecological succession are more limited by N availability when compared with trees in primary forest (Zhang et al., 2015). Therefore, the most important aspect of using shrubs successfully during habitat restoration may be their capacity to grow under low nutrient conditions and increase soil N availability when their biomass is turned over.

Oxalic acid is a small component of organic acids, which exude from roots and hyphae and have been suggested to play an important role in enhancing nutrient acquisition by plants in calcareous soils (Strom et al., 2001, 2005; Tu et al., 2007; Clarholm et al., 2015). Clarholm et al. (2015) have suggested that oxalic acid destabilizes the Ca bridges within SOM in the rhizosphere soil, while Strom et al. (2001, 2005) documented the same process to be active between SOM and mineral surfaces. The breaking of Ca bridges opens up secondarily built SOM supramolecules aggregates (Simpson et al., 2002) so that new surfaces will be locally exposed for soil microbes to attack with their extracellular enzymes and thus locally enhance N release from SOM (Phillips et al., 2011). The released N is rapidly absorbed by fine roots. Despite many observations of oxalic acid enhancing N availability (Griffiths et al., 1994; Strobel, 2001; Camps Arbestain et al., 2003), little information is available about how oxalic acid enhances soil N availability in karst ecosystems, and also elsewhere. We decided to test the model suggested by Clarholm et al. (2015) in a shrubland on deteriorated and formerly deforested land that had undergone natural restauration for almost 25 years and in a primary forest that had been undisturbed for 200 years.

In the studied karst ecosystem two shrub species (*Alchornea trewioides* and *Ligustrum sinense*) and two tree species (*Celtis biondii* and *Pteroceltis tatarinowii*) were selected as research subjects; these are all native plant species in this region (Zhang et al., 2010; Liu et al., 2011; Wang and Xu, 2013; Pan et al., 2015). The selected shrubs species were limited by N and P while the trees species were mainly limited by P in karst ecosystems (Zhang et al., 2015). To explore the effects of oxalic acid on N availability for these species with different nutrient limitations, oxalic acid, microbial biomass carbon (MBC), β-1,4-*N*-acetylglucosaminidase (NAG), and potential N mineralization rates were measured in the rhizosphere of four plant species in both non-growing and growing seasons. Our hypothesis was that we should be able to demonstrate a positive relationship between concentrations of oxalic acid and the three other parameters.

## Materials and Methods

### Study Site

The study was conducted at the Huanjiang Observation and Research Station for Karst Ecosystems of the Chinese Academy of Sciences (24°43″58.9″–24°44′48.8″N, 108°18′56.9″–108°19′58.4″E) and the Mulun National Natural Reserve (25°06′–25°12′ N, 107°53′–108°05′E); both sites are in Huanjiang County, Guangxi Zhuang Autonomous Region, in southwestern China. Both sites experience a typical subtropical monsoon climate with a mean annual temperature of approximately 19°C and precipitation of 1400–1500 mm, most of which falls from May to September (Chen et al., 2010; Peng et al., 2012). The typical karst landscape of both sites lies in a gentle valley flanked by steeper hills. Soil pH at the study sites ranged from 6.3 to 7.9. Soil depth averaged 50–80 cm in valleys and 10–30 cm on hillslopes.

The Huanjiang Observation and Research Station covers an area of 146 km^2^ and contains 7.5 km^2^ of farmland that is mainly located in the valley. This area experienced severe deforestation from 1958 to the mid-1980s, and natural restoration has been ongoing for almost 25 years. Currently, shrubs dominate about 70% of the hillslopes and commonly include *A*. *trewioides*, *Cipadessa baccifera*, *Indigofera atropurpurea*, *L. sinense*, and *Rhus chinensis* (Nie et al., 2012). The Mulun National Natural Reserve, approximately 35 km from northwest of the Huanjiang Observation and Research Station, covers an area of 108.6 km^2^ and was established in 1991 to protect a remnant of undisturbed mixed evergreen and deciduous broadleaved forest in the karst region. The primary forest on this reserve has not been disturbed for over 200 years and supports the dominant species of *C. biondii*, *Cleidion bracteosum*, *Cryptocarya chinensis*, *Cyclobalanopsis glauca*, *Loropetalum chinense*, *Miliusa chunii*, and *P. tatarinowii*; these are typical plant species of climax communities in this subtropical karst region (Su and Li, 2003; Pan et al., 2015).

### Sampling

Two dominant shrubs species (*A. trewioides* and *L. sinense*) of these shrublands were selected, and two dominant trees (*C. biondii* and *P. tatarinowii*) of primary forests were selected for comparison. Rhizosphere soil sampling was conducted in January (mid-non-growing season, during the dry season) and July (mid-growing season, during the wet season) 2014. Three types of slope position (i.e., lower, middle, and upper slopes) were selected to exclude the effects of slope position on the concentrations of oxalic acid, MBC, and NAG as well as potential N mineralization rates (Stewart et al., 2014). Additionally, to minimize the effects of interactions that might occur between hosts, plants were separated by approximately 50 cm in each slope position. A total of 90 soil samples [i.e., four plant species × three slope positions × two seasons × four (three if we could not find a fourth individual at a particular site) replicate samples] were collected.

The rhizosphere soil was defined as the soil that remained and adhered to the surface of roots after loose soil had been shaken off (Bell et al., 2014). First, the fine roots and soils were dug out from the topsoil at 0–15 cm depth. Second, the roots with soil were lightly and carefully shakened to remove loose soil. Then, the rhizosphere soil of each individual plant of each species was carefully removed as a separate sample from the surface of the roots (Macrae et al., 2001). Each sample of collected soil was then placed into a separate polyethylene bag, put in an ice box, and carried to the laboratory; fine roots were cleared within 4 h of collection in the laboratory. Next, each sample was divided into three equal parts. One part was stored at -20°C for oxalic acid and enzyme concentration assay; one part was stored at 4°C for MBC concentration and potential N mineralization rate assay; and one part was air dried, ground to pass a 2-mm mesh sieve for soil chemical analysis (**Table 1**).

**Table 1:** Characteristics of rhizosphere soils of the four plant species in karst ecosystems of southwest China (mean ± SE).

Source of rhizosphere soil	SOM	TN	TP
*Alchornea trewioides* (shrub)	129.11 ± 4.30a	4.77 ± 0.21a	1.38 ± 0.04a
*Ligustrum sinense* (shrub)	114.06 ± 5.82a	4.72 ± 0.38a	1.37 ± 0.07a
*Celtis biondii* (tree)	203.74 ± 14.84b	9.98 ± 0.71b	1.54 ± 0.09a
*Pteroceltis tatarinowii* (tree)	186.14 ± 15.55b	8.50 ± 0.75b	1.57 ± 0.10a
Shrubs (mean of two species)	121.59 ± 5.06A	4.75 ± 0.30A	1.38 ± 0.05A
Trees (mean of two species)	194.94 ± 15.20B	9.24 ± 0.73B	1.56 ± 0.09A

*SOM, soil organic matter (g. kg^-1^ soil); TN, total nitrogen (g. kg^-1^ soil); TP, total phosphorus (g. kg^-1^ soil). Small letters in each column indicate significant differences (p < 0.05) among four plant species; capital letters indicate significant differences (p < 0.05) between shrubs and trees.*

### Laboratory Analyses

#### Oxalic Acid Concentrations

To determine the oxalic acid concentration, first, sample suspensions were prepared by adding 2.5 g of soil to 5 mL of 0.05 mol/L phosphate buffer. Next, samples were homogenized for 10 min with a homogenizer, and were then centrifuged for 10 min at 15,000 rpm by an ultracentrifuge (CR22 GII; Hitachi Koki Co., Ltd, Japan). The liquid supernatant from each sample was purified by being passed through a 0.22-μm filtering membrane. The oxalic acid concentration of the purified liquid was determined using high-performance liquid chromatography (1260 Infinity LC; Agilent Technologies, Santa Clara, CA, USA) (Kurosumi et al., 2008).

#### NAG Activities

We measured the activity of a hydrolytic enzyme (NAG) involved in the decomposition of N from SOM (Finzi et al., 2006). Sample suspensions were prepared by adding 1 g of soil to 125 mL of 50 mM, pH5.0 acetate buffer and homogenizing for 1 min with a homogenizer. The suspensions were stirred and eight replicates were produced; 200 μL aliquots were dispensed into 96-well microplates. Fifty microliters of 200 μL 4-methylumbelliferone solution was added to each well. Overall, there were eight replicate wells for each blank, negative control, and quench standard. Microplates were incubated in the dark at 20°C for 2 h. To stop the reaction, a 10-μL aliquot of 1.0 M NaOH was added to each well. NAG activity was measured by fluorescence using 4-methylum-belliferyl *N*-acetyl-β-D-glucosaminide as a substrate and a microplate fluorometer (Infinite M200 PRO; TECAN, Switzerland) with 365 nm excitation and 450 nm emission filters. After correction for controls and quenching, NAG activity was expressed in units of μmol g^-1^soil h^-1^.

#### Microbial Biomass C

The soil MBC analyses were conducted by chloroform fumigation-K_2_SO_4_ extraction carbon automatic analysis (Wu et al., 1990). Total organic C was extracted with K_2_SO_4_ (10 g of soil in 40 mL of 0.5 mol.L^-1^ K_2_SO_4_) under fumigated and unfumigated status. Fumigated and unfumigated extracts were analyzed for total extractable organic C using a total organic carbon analyzer (Shimadzu TOC-Vwp; Shimadzu Corporation, Kyoto, Japan).

#### N Mineralization Potentials

Potential N mineralization rates were measured by laboratory incubation. Inorganic N (NH4+) was extracted immediately from soils with 2 M KCl and measured with a FIAstar 5000 flow injection analyzer (FOSS, Hillerød, Denmark). Potential mineralization rates were estimated by extracting soil inorganic N from samples after incubating soils aerobically in the laboratory at 25°C for 15 days; soil moisture was not adjusted prior to the incubations, and soils were incubated in plastic cups covered with punctured plastic wrap to minimize soil moisture loss while allowing for aeration (Phillips et al., 2012).

#### Soil Chemical Properties

Soil organic matter concentrations were measured using wet oxidation by KCr_2_O_7_ + H_2_SO_4_ and titration with FeSO_4_. Total soil N (TN) was measured with a FIAstar 5000 FOSS using the Kjeldahl method. Total soil P (TP) was digested in a solution of H_2_SO_4_ + HClO_4_ and analyzed with blue phosphor-molybdate, and available phosphorus (AP) was measured as soil P after extraction with NaHCO_3_ (Liu et al., 2015b).

### Statistical Analyses

**FIGURE 1:**
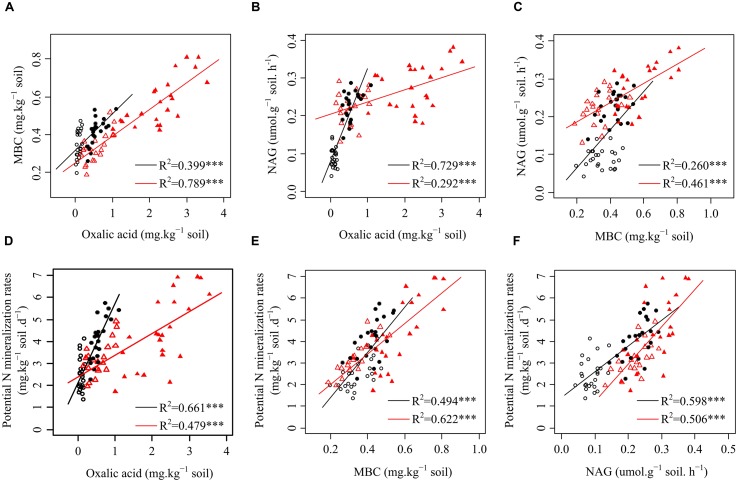
**Relationships among oxalic acid, microbial biomass carbon (MBC), β-1,4-*N*-acetylglucosaminidase (NAG) activity, and potential N mineralization rates of the four plants in karst ecosystems of southwest China. (A)** Relationship between oxalic acid and MBC of shrubs and trees. **(B)** Relationship between oxalic acid and NAG of shrubs and trees. **(C)** Relationship between MBC and NAG of shrubs and trees. **(D)** Relationship between oxalic acid and potential N mineralization rates of shrubs and trees. **(E)** Relationship between MBC and potential N mineralization rates of shrubs and trees. **(F)** Relationship between NAG and potential N mineralization rates of shrubs and trees. ○ shrubs, non-growing season; ● shrubs, growing season; △ trees, non-growing season; ▲ trees, growing season; black and red regression lines are for shrubs and trees, respectively; ^∗∗∗^*p* < 0.001.

**FIGURE 2:**
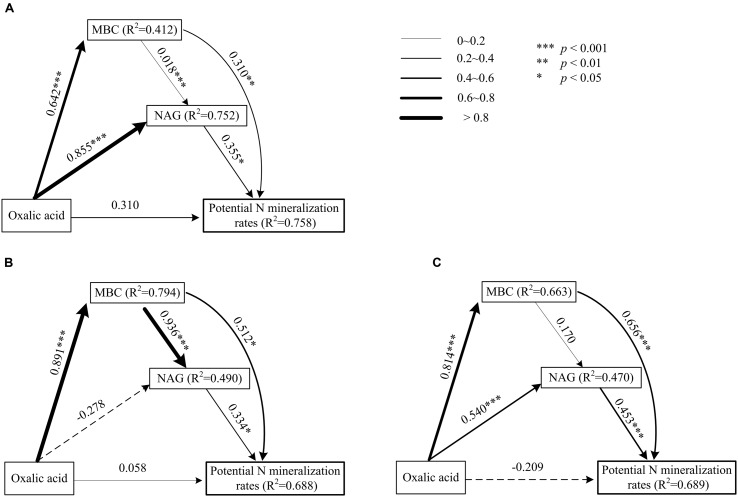
**Path model of direct and indirect effects of oxalic acid, MBC, and β-1,4-NAG activity on potential N mineralization rates of shrubs (A), trees (B), and two functional groups (C) in karst ecosystems, based on Clarholm et al. (2015) about the mechanism of enhancing nutrient acquisition for plants.** Each value associated with an arrow is a path coefficient and is equivalent to the direct effect of the independent varible on the dependent variable.

Data treatment and calculations were performed using the R x64 3.2.0 statistical software (R Core Team, 2015). The homogeneity of variance was tested prior to the statistical analysis. First, the effects of plant species and functional groups on the variance of rhizosphere oxalic acid, MBC, NAG, and potential N mineralization rates in both seasons were analyzed with linear mixed models (lme4 package with R) (Pan et al., 2015). The plant species and functional groups were modeled as fixed factors with site as a random factor (**Table 1**). The level of significance was defined as *p* < 0.05. We then built linear regression models to quantify the relationships among oxalic acid, MBC, NAG, and potential N mineralization rates for each plant functional group and present them as scatter plots (**Figure 1**). Path analysis (plspm package with R) was applied to investigate the direct and indirect effects of oxalic acid, MBC, and NAG on potential N mineralization rates of shrubs (**Figure 2A**), trees (**Figure 2B**), and overall functional groups (**Figure 2C**), based on Clarholm et al. (2015) related to the mechanism of enhancing nutrient acquisition for plants. We calculated the standardized total effects (direct plus indirect effects from the path analysis model) of oxalic acid, MBC, and NAG on potential N mineralization rates (Delgado-Baquerizo et al., 2016).

## Results

### Patterns of Oxalic Acid, MBC, NAG, and Potential N Mineralization Rates

Plant species and plant functional groups significantly affected the oxalic acid concentrations, amounts of MBC, NAG activities, and potential N mineralization rates (**Table 2**). The oxalic acid concentrations, amounts of MBC, NAG activities, and potential N mineralization rates in rhizosphere soils of the shrub *A. trewioides* were the lowest in both seasons among the four plant species. In both seasons, the oxalic acid concentrations and NAG activities were both significantly lower in rhizosphere soils of shrubs (*A. trewioides* and *L. sinense*) than of trees (*C. biondii* and *P. tatarinowii*). The amounts of MBC in rhizosphere soils of shrubs were significantly lower than that of trees during the growing season, but the measure of shrubs and trees were similar in the non-growing season. The potential N mineralization rates showed a dissimilar pattern of MBC where the potential N mineralization rates in rhizosphere soils of trees were significantly higher than that of shrubs in the non-growing season but similar in the growing season.

**Table 2:** Oxalic acid (mg. kg^-1^ soil), microbial biomass (MBC; mg kg^-1^ soil), β-1,4-*N*-acetylglucosaminidase activity (NAG, μmol g^-1^ soil h^-1^), and potential N mineralization rates (mg kg^-1^ soil d^-1^) in rhizosphere soils of the four plant species and two functional groups in karst ecosystems of southwest China (mean ± SE).

	Oxalic acid	MBC	NAG activity	Potential N mineralization rates
**Non-growing season**				
*Alchornea trewioides* (shrub)	0.04 ± 0.00a	0.323 ± 0.022a	0.094 ± 0.009a	2.61 ± 0.22a
*Ligustrum sinense* (shrub)	0.14 ± 0.01b	0.367 ± 0.019a	0.085 ± 0.006a	2.36 ± 0.21a
*Celtis biondii* (tree)	0.50 ± 0.13c	0.343 ± 0.014a	0.253 ± 0.011c	3.53 ± 0.22b
*Pteroceltis tatarinowii* (tree)	0.59 ± 0.07c	0.323 ± 0.029a	0.202 ± 0.012b	3.01 ± 0.24a
Shrubs (mean of two species)	0.09 ± 0.01A	0.345 ± 0.015A	0.090 ± 0.005A	2.49 ± 0.15A
Trees (mean of two species)	0.56 ± 0.07B	0.331 ± 0.018A	0.222 ± 0.010B	3.22 ± 0.18B
**Growing season**				
*Alchornea trewioides* (shrub)	0.44 ± 0.02a	0.396 ± 0.023a	0.225 ± 0.011a	4.02 ± 0.19a
*Ligustrum sinense* (shrub)	0.66 ± 0.07b	0.442 ± 0.015a	0.231 ± 0.012a	4.23 ± 0.37a
*Celtis biondii* (tree)	2.44 ± 0.26c	0.606 ± 0.047b	0.314 ± 0.013b	4.94 ± 0.49a
*Pteroceltis tatarinowii* (tree)	2.13 ± 0.13c	0.542 ± 0.025b	0.244 ± 0.016a	3.92 ± 0.45a
Shrubs (mean of two species)	0.55 ± 0.04A	0.418 ± 0.015A	0.228 ± 0.008A	4.12 ± 0.20A
Trees (mean of two species)	2.28 ± 0.14B	0.573 ± 0.026B	0.277 ± 0.012B	4.41 ± 0.34A

*Small letters in each column indicate significant differences (p < 0.05) among four plant species; capital letters indicate significant differences (p < 0.05) between shrubs and trees.*

### N Availability Correlated with Oxalic Acid, MBC, and NAG

Significantly positive relationships were observed among oxalic acid concentrations, amounts of MBC, NAG activities, and potential N mineralization rates for both shrubs and trees (**Figure 1**). However, the relationships between oxalic acid concentrations, amounts of MBC, NAG activities, and potential N mineralization rates were affected by functional group because the slopes and intercepts were different for shrubs and trees for all four investigated parameters (**Figures 1A–F**). Path analysis showed that an increased oxalic acid concentration had a positive effect on amounts of MBC in shrubs, trees, and overall functional groups and on NAG activities in shrubs and all species of overall functional groups, while it had no direct effect on potential N mineralization rates (**Figure 2**). Additionally, the MBC and NAG directly affected potential N mineralization rates in shrubs (**Figure 2A**), trees (**Figure 2B**), and overall functional groups (**Figure 2C**).

## Discussion

### Oxalic Acid Enhancement of N Availability as a Main Nutrient-Acquisition Strategy for Plants in Karst Regions

In a karst ecosystem, a high CaCO_3_ concentration in soil has a strong effect on stabilizing SOM (Hu et al., 2012; Clarholm et al., 2015), which leads to low amounts of C, N, and P being released from SOM. In the present study, higher concentrations of total soil N were observed in rhizosphere soils of trees than shrubs, while low concentrations of total soil P were found in both treatments (**Table 1**). This outcome was in agreement with a previous study by our team that plants growing during the early stage of ecological succession (such as shrubland) are mainly limited by N and P, while plants in a later stage of ecological succession (such as primary forest) are limited by P (Zhang et al., 2015). One possible explanation for this observation is that the trees takes up Ca from deep soil, resulting in increasing pH and Ca incorporation in SOM (Clarholm and Skyllberg, 2013). However, the shrubland analyzed in the present study is experiencing ecological succession into a forested condition, and is exhibiting a relatively low nitrogen concentration, pH (the mean values in shrubland and primary forest is 6.8 and 7.1, respectively), and SOM (Liu et al., 2015b).

To enhance nutrient acquisition from SOM, plants may be able to stimulate microbial interactions with SOM. A three-step process has been suggested (Clarholm et al., 2015), and this process was tested here. Organic acids exuded into soils by roots, mycorrhizal fungi or bacteria react either with polyvalent metal bridges in SOM or with chemical bindings between SOM-CaCO_3_. This destabilizes the SOM supramolecule aggregates and leads to exposition of protected areas of good quality. Microorganisms send out a small but continuous flow of exteracellular enzymes (especially NAG enzyme) into their environment to test it. If these enzymes meet with newly exposed surface areas and manage to release N molecules, which are diffusing back into the microorganisms, then the microbes will rapidly increase their enzyme production. As a result, fine roots and symbiotic fungi are able to achieve nutrient uptake. The three-step process indicates that oxalic acid indirectly affected potential N availability. This model was nicely supported by our observations from rhizosphere soils of karst plant species. Based on path analysis, our results showed that increased levels of oxalic acid can enhance potential N mineralization rates through inducing microbial and NAG activities (**Figure 2**). Oxalic acid enhancement of N availability can therefore be considered to be a nutrient-acquisition strategy of plants in karst regions.

### Factors Driving the Observed Differences in Oxalic Acid Concentration

**FIGURE 3:**
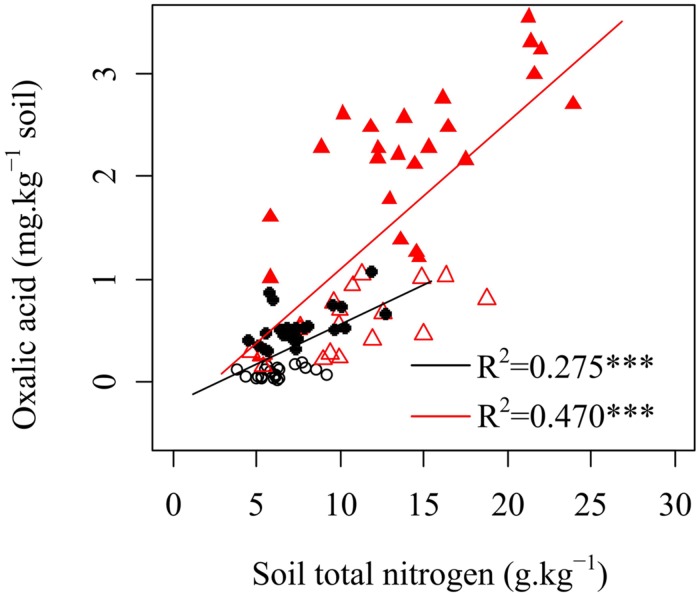
**Relationships between oxalic acid and soil total nitrogen in rhizosphere soils of shrubs and trees.** ○ shrubs, non-growing season; ● shrubs, growing season; △ trees, non-growing season; ▲ trees, growing season; black and red regression lines are for shrubs and trees, respectively; ^∗∗∗^*p* < 0.001.

Many studies have shown that when in soils with limited N content, plants increase N availability by enhancing root exudation (Fontaine et al., 2011; Yoneyama et al., 2013). The mechanisms following the increased root-derived exudation are not yet well understood. It should be a mechanism where exudation enhance the activities of microorganisms (mainly fungi) and related enzymes activity in a way that accelerates SOM decomposition and increased N availability for plants (de Graaff et al., 2009; Phillips et al., 2011; Bengtson et al., 2012; Drake et al., 2013; Finzi et al., 2015). Thus, soil fertility should be an important factor that influences the changes in oxalic acid concentration. In our study, oxalic acid concentrations were positively correlated with total soil N (**Figure 3**), which was inconsistent with these previous studies. Several potential explanations can be proposed for this result. First, plants will reduce their exudation under nutrients deficiency because the exudations (mainly organic C) may cause plants to lose energy and material (Zhu et al., 2014). Oxalic acid, that provides only a small part of total exuded C, would increase with increasing total exuded C as conditions change from nutrient-poor to nutrient-rich soils. Second, mycorrhizal fungi help karst plants absorb a large amount of N from soil. In nutrient-poor soil, a diverse species assemblage and a large population of arbuscular mycorrhizal fungal species has been observed to meet the demand for nutrients required for plant growth (Johnson et al., 2010). A previous study in the present region has shown that the Shannon–Wiener diversity of arbuscular mycorrhizal fungal community decreases but total soil nitrogen concentration increases along a gradient of natural vegetation successional stages (grassland, shrubland, secondary forest, and primary forest; Liang et al., 2015). This finding suggests that the presence of more complex arbuscular mycorrhizal fungal assemblages might reduce the N limitation of shrubs and improve N uptake of plants when the total soil N concentration is low. Soils with higher nutrient levels and pH support larger bacterial populations, but have fewer fungal hyphae. Thus, the positive relationship between oxalic acid and soil total N concentration (**Figure 3**) suggested that oxalic acid exudation by plants could indirectly increase organic N in karst ecosystems via their biomass production. Oxalic acid is short-lived and becomes undetectable as soon as it reacts with its environment. Microbes and enzymes are both more long-lived in soil than organic acids. Potential N mineralization measured with the 15 days incubation is measuring the history of N levels. It has been suggested to picture the amount of microorganisms turned over the past growing season, a fraction more available for N mineralization than N in SOM as a whole.

Plants should have a fundamental role in driving the changes in oxalic acid concentration in soils. In the present study, the oxalic acid concentrations and potential N mineralization rates varied among the four plant species (**Table 2**). The oxalic acid concentrations and potential N mineralization rates in rhizosphere soils of the two shrubs (*A*. *trewioides* and *L. sinense*) were significantly lower than those of the two trees (*C. biondii* and *P*. *tatarinowii*) in both seasons (**Table 2**). These results indicated that over time the pattern of oxalic acid concentration should be based on the physiological needs of different plants for growth. The lower net primary production of shrubs than trees (Zhou et al., 2014) suggests that the lower photosynthetic potential and lower N requirements of shrubs allow this ecosystem to support their growth (Pan et al., 2015). Our observations support this conclusion.

**FIGURE 4:**
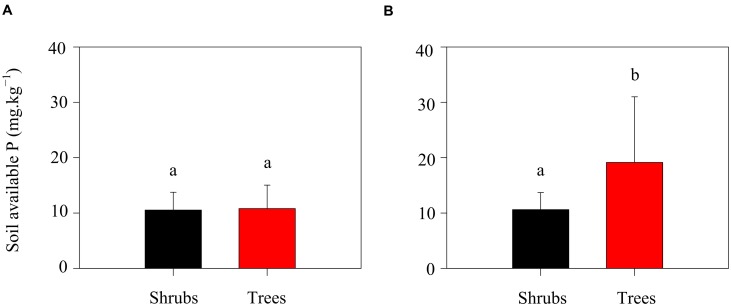
**Soil available P of shrubs and trees in the non-growing (A) and growing (B) seasons.** Different letters above error bars indicate significant differences between shrubs and trees at *p* < 0.05.

Season did also influence the level of oxalic acid concentration. Oxalic acid concentrations were higher during the growing season than that during the non-growing season (**Table 2**). In the growing season, high temperatures and precipitation would benefit plant growth and thus stimulate exudation of oxalic acid. Simultaneously, the higher microbial activity in the growing season would also increase the exudation of oxalic acid. Additionally, the potential N mineralization rates of the two shrubs were slightly lower than those of trees during the growing season, while the oxalic acid concentration was four times higher in the rhizosphere of trees than in that of shrubs (**Table 2**). One possible explanation for this observation may be related to the availability of P for trees growth. In this ecosystem, calcium-rich soils may compete with plants for available P (Vitousek et al., 2010). Thus, trees are constrained by P limitation in this region (Zhang et al., 2015). The relatively low P concentrations in litters when compared with soils (Pan et al., 2011) suggests that enzymatic release of P from plant litters might be more difficult to perform than from soil substrates. Oxalic acid is a major factor that enhances P availability in these ecosystems (Strom et al., 2005) because it can destabilize the molecular bridge in P-Ca compounds (Clarholm et al., 2015). To increase P supply during the growing season, trees would exudate greater amounts of oxalic acid leading to increased higher amounts of available P in the rhizosphere soils of trees when compared with shrubs (**Figure 4**). Trees obviously exude oxalic acid to increase both N and P availability. In contrast, no significant correlation between available P and oxalic acid concentration in shrubs was found in the present study (**Figure 4**). Shrubs should exude relatively lower amounts of oxalic acid to support their lower growth rates when compared with trees.

Based on our observations, increasing the oxalic acid concentration of rhizosphere soil is believed to be a good method for supporting successful vegetation restoration in karst regions. Exudation rates of oxalic acid in dominant shrub plants should be determined by field surveys, and plants with high exudation rates should be selected for shrub vegetation restoration. Alternatively, oxalic acid could also be directly added into soils to increase nutrient availability for plant growth because of its positive effect on plant growth (Nikbakht et al., 2008). Hence, the effects of oxalic acid on plant growth should be well quantified in further studies so that its use can be improved during vegetation restoration in karst regions.

## Conclusion

In this study, we provide new insights into the availability of nutrients for shrub and tree species in karst regions while considering the related biochemical situation. The potential N mineralization rates as well as oxalic acid concentration, MBC, and NAG activity in rhizosphere soils varied among species, functional groups, and seasons. Potential N mineralization rates were directly affected by MBC and NAG, but were only indirectly affected by oxalic acid concentration. However, MBC and NAG were directly affected by oxalic acid concentration. This indicated that oxalic acid increased potential N mineralization rates mainly through exposing new surfaces of SOM, in turn enhancing microbial and NAG activities in the rhizosphere. The results suggested that the exudation of oxalic acid is an important nutrient-acquisition strategy of plants in karst ecosystems, as well as in other nutrient-limited ecosystems.

## Author Contributions

FP, YL, WZ, and KW designed the experiments; FP performed the experiments; FP and YL carried out data analysis and wrote the manuscript; WZ, JZ, and KW helped in interpretation of the analyses during constructive discussions.

## Conflict of Interest Statement

The authors declare that the research was conducted in the absence of any commercial or financial relationships that could be construed as a potential conflict of interest.
